# Spontaneous autoimmune subepidermal blistering diseases in animals: a comprehensive review

**DOI:** 10.1186/s12917-023-03597-1

**Published:** 2023-02-27

**Authors:** Petra Bizikova, Thierry Olivry, Keith Linder, Jan Rybnicek

**Affiliations:** 1 Department of Clinical Sciences, College of Veterinary Medicine, North Carolina State University, 1060 William Moore Drive, Raleigh, NC 27607 USA; 2 Department of Population Health and Pathobiology, College of Veterinary Medicine, North Carolina State University, Raleigh, NC USA; 3 Veterinary Dermatology and Dermatopathology Service, Padochov 175, 66491 Ivancice, Czech Republic

**Keywords:** Autoimmune, Skin, Pemphigoid, Dog, Cat, Blister

## Abstract

Autoimmune subepidermal blistering diseases (AISBDs) are rare skin disorders of animals that were first identified in dogs but several AISBDs are now recognised in other companion animal species. Most AISBDs in animals are homologues of the human diseases and are thought to share similar pathomechanisms of epidermal and/or mucosal blister formation caused by autoantibodies targeting structural proteins of the basement membrane zone (BMZ). Disruption of their structural function by the autoantibodies and/or recruited inflammation leads to BMZ fragility, which presents clinically as vesicles, bullae and, later, deep erosions and ulcers. Canine AISBDs are the best characterised, particularly the more common variants such as mucous membrane pemphigoid (48%), epidermolysis bullosa acquisita (EBA) (26%), and bullous pemphigoid (10%). Exceedingly rare AISBDs in the dog are junctional EBA, mixed AISBD, type-1 bullous systemic lupus erythematosus, linear IgA dermatosis, and pemphigus gestationis. The diagnosis of a specific AISBD is made by combining the clinical features (breed, age, lesion distribution) with histological evidence of subepithelial clefting, but not all AISBDs can be differentiated in this manner and specialised immunological testing is required. This latter, unfortunately, is not readily available and, therefore, the specific AISBD diagnosis often remains unconfirmed. While this limits further understanding of these diseases, it does not prevent clinicians from treating their patients, as the treatment approaches are similar for the different AISBDs in dogs. This review primarily focuses on canine AISBDs, the species for which these diseases have been best characterised, and shorter descriptions of variants in other species are also provided.

**Supplementary Information:**

The online version contains supplementary material available at 10.1186/s12917-023-03597-1.

## Introduction

**Fig. 1:**
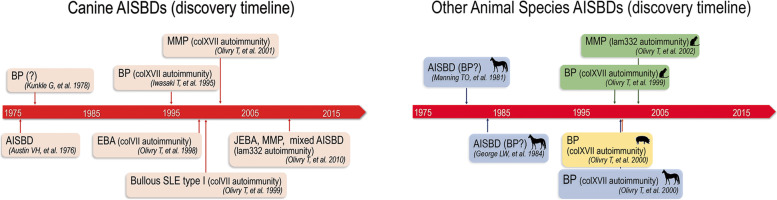
Autoimmune subepidermal blistering diseases – Discovery timeline. Abbreviation: AISBD autoimmune subepidermal blistering disease, BP bullous pemphigoid, bullous SLE bullous systemic lupus erythematosus, EBA epidermolysis bullosa acquisita, JEBA junctional epidermolysis bullosa acquisita, MMP mucous membrane pemphigoid

**Fig. 2:**
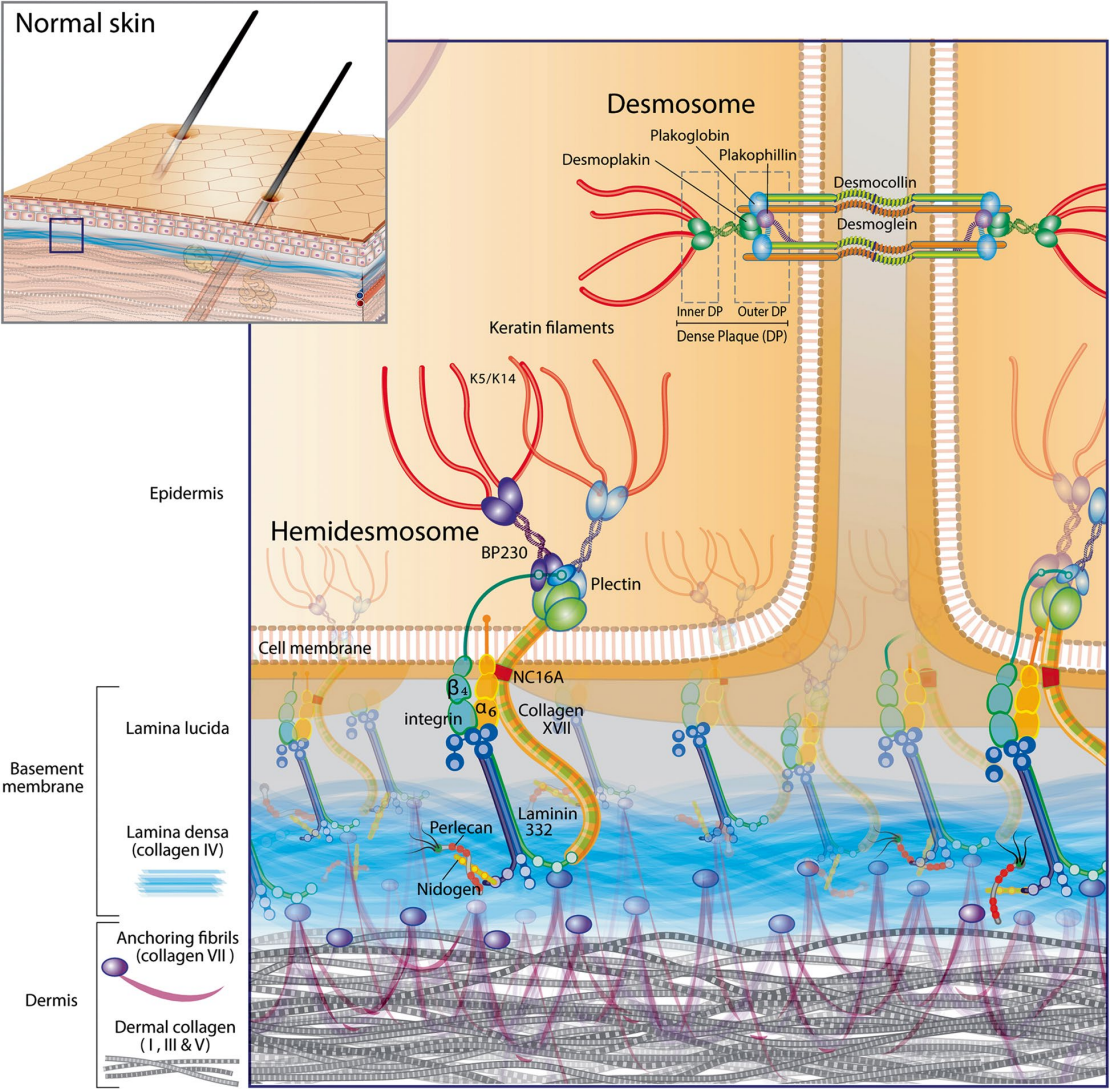
Basement membrane zone diagram. Artist: Alice Harvey

Autoimmune subepidermal blistering diseases (AISBDs) are rare skin disorders of animals that were first identified in dogs before being described, even more rarely, in other companion species. Their first description in companion animals was made in a dog more than 40 years ago [1]. Between the late 1970s and 1995, most of the dogs with histological evidence of a subepidermal blister formation were given the diagnosis of bullous pemphigoid (BP); a diagnostic approach that is no longer supported using today’s criteria (reviewed in [2]). It was not until the emergence of more advanced laboratory techniques in veterinary medicine that the identification of the individual AISBD variants in dogs was made (Fig. 1; Supplemental Table 1) [2–7]. Most of these diseases are homologues of human diseases; some have been recognised in other animal species as well (Fig. 1; Supplemental Table 1) [8–13]. The common immune mechanism shared by these diseases is an autoantibody response directed against structural proteins of the dermo-epidermal junction (i.e., the epidermal basement membrane; Fig. 2) resulting in dermo-epidermal blister formation. Multiple pathomechanisms have been proposed to cause this dermo-epidermal separation. The humoral immune response, in conjunction with complement activation, neutrophil and/or eosinophil recruitment and Fc-receptor mediated inflammation, has been shown to have damaging effects on basement membrane zone (BMZ) structures in several human AISBDs [14–17]. Furthermore, complement-independent pathogenic effects of autoantibodies, IgG4 particularly, have been demonstrated in some disease models [15, 16]. Because of the similarities, the pathomechanism(s) of blister formation in veterinary species are presumed to share features of their human counterparts; although, they have not been investigated yet.

**Fig. 3:**
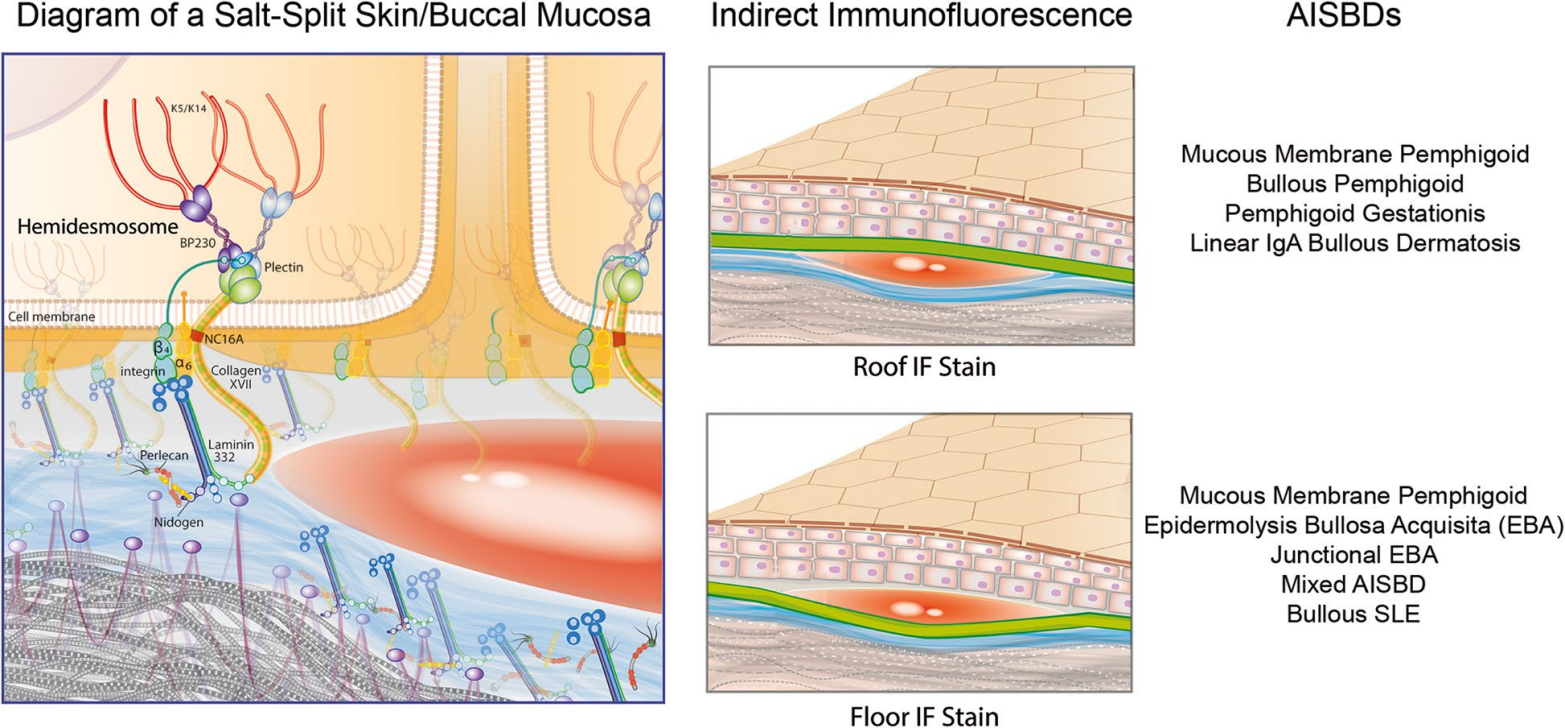
Salt-split skin substrate, individual indirect immunofluorescence staining patterns and corresponding autoimmune subepidermal blistering diseases. Abbreviations: AISBD autoimmune subepidermal blistering disease, bullous SLE bullous systemic lupus erythematosus. Note: Mixed dermal and epidermal immunofluorescence staining pattern can be seen in some AISBDs (e.g., junctional EBA; discussed further in the text). Artist: Alice Harvey

**Fig. 4:**
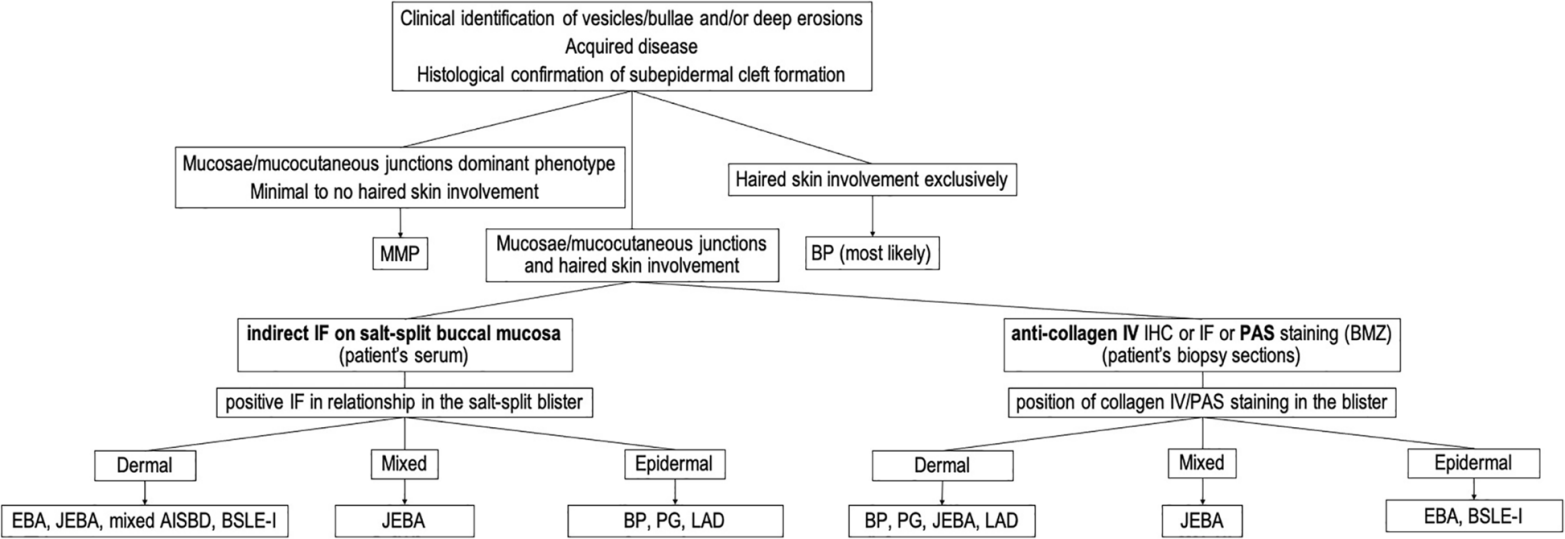
Autoimmune subepidermal blistering diseases – Diagnostic diagram

The diagnosis of a specific AISBD is usually made by combining clinical features with histological and, depending on the disease and availability of specialised laboratory testing, immunopathological data (e.g., direct immunofluorescence (IF), indirect IF on salt-split skin (Fig. 3), antigen-specific ELISA). A simplified diagnostic algorithm using diagnostic tests potentially accessible to veterinarians is depicted in Fig. 4. Unfortunately, commercial laboratories do not offer routine immunological testing for veterinary species and, therefore, the diagnostic ability of veterinarians to fully confirm their diagnosis is somewhat limited. To overcome these limitations, veterinarians often use a periodic acid-Schiff stain (PAS) or anti-collagen IV immunohistochemistry (IHC) to demonstrate the level of the dermo-epidermal split to narrow down the list of possible AISBD variants. Because of their frequently negative staining, these techniques have been shown unreliable for distinguishing individual AISBDs in people [18]. As a result, several recently published cases in the veterinary literature have lacked advanced immunotesting (i.e., antigen confirmation) and are thus reported as having AISBDs without further specification [19–22].

This review primarily focuses on canine AISBDs, the species for which these diseases have been best characterised; shorter descriptions of the variants in other species are made, where needed. The individual diseases are organised based on their prevalence in dogs rather than the target antigens, which are listed in Supplemental Table 1. A note is made at the end of each section to indicate other species in which the particular disease has been recognised.

## Autoimmune subepidermal blistering diseases

## Mucous membrane pemphigoid

Previously known as “cicatricial pemphigoid”, the consensus terminology of human mucous membrane pemphigoid (MMP) was proposed by Chan and his colleagues in 2002 [23]. Since then, MMP represents an immunologically heterogeneous disease with lesions predominantly affecting mucosae and mucocutaneous junctions [23]. First “officially” reported as an individual entity in dogs in 2001 [6], naturally occurring MMP has been described also in cats [10, 13, 24]. Relevant information on canine and feline MMP has been extracted from a previously published meta-analysis (42 dogs; 1970–2002) [25], one case series (16 dogs; 2003–2014) [26] and recent case reports describing dogs and cats with AISBD with a mucosae/mucocutaneous junction-predominant phenotype (4 dogs, 4 cats) [7, 10, 13, 20, 21, 24, 27]. Altogether, we report data on 62 dogs and four cats with MMP.

### Canine mucous membrane pemphigoid

#### Prevalence and signalment

Mucous membrane pemphigoid is the most common AISBD recognised in dogs (48% of all AISBDs [2]). It occurs equally between females and males (M:F ratio 1.1) and the German shepherd dog and its crosses appear to be overrepresented (18/62; 29%). Other reported breeds with more than three cases per breed include collies, shelties and Australian shepherds (6; 10%), poodles (5; 8%), cocker and springer spaniels (5; 8%) and Siberian husky crosses (3; 5%). The median age of onset is 5 years (range: 1–15 years), although almost one third of dogs (28%) are 8 years or older at the time of the disease onset.

#### Clinical signs

**Fig. 5:**
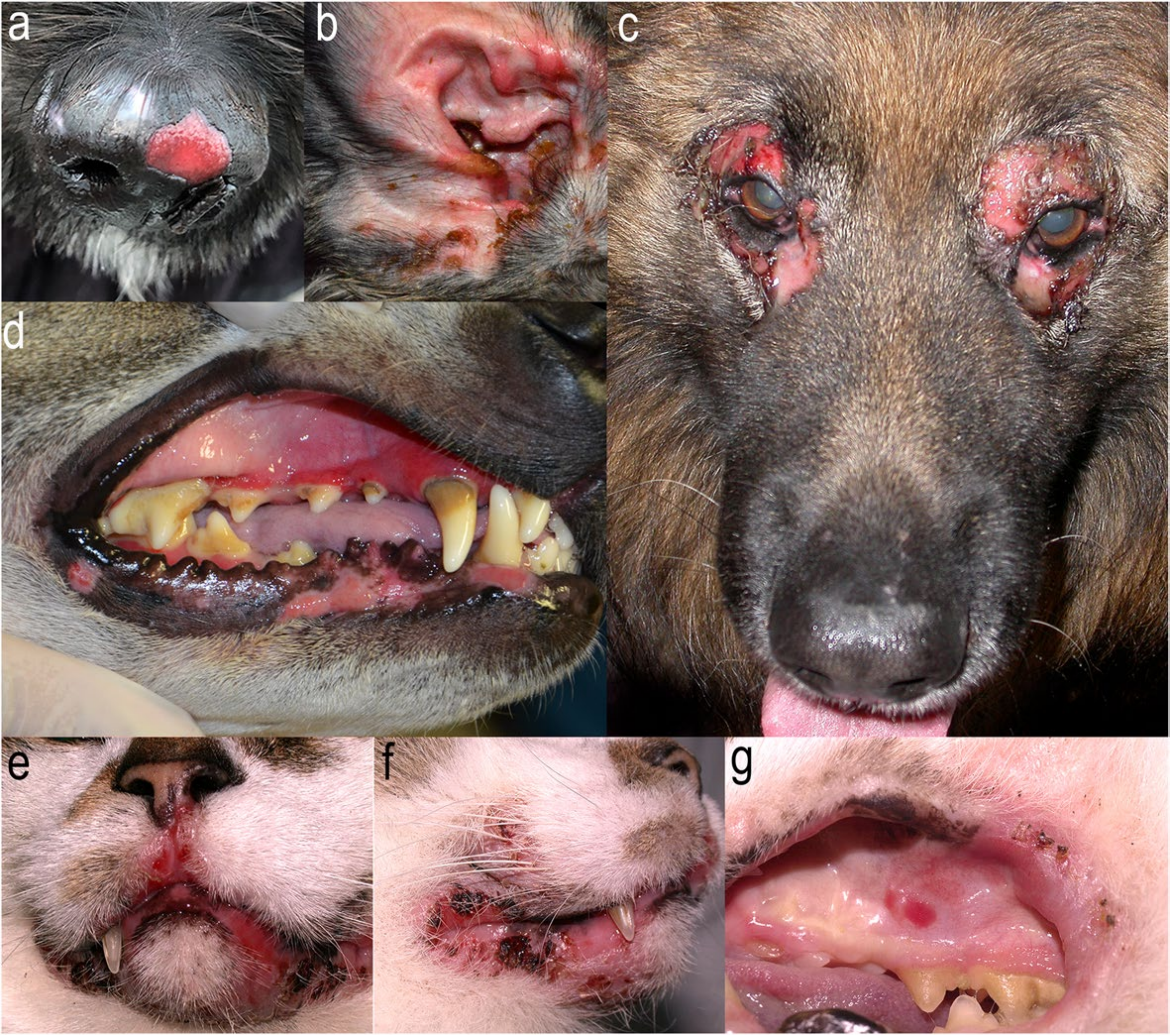
Canine (**a**-**d**) and feline (**e**-**f**) mucous membrane pemphigoid: Deep erosion with a partial remnant of a recently ruptured bulla (**a**), deep erosions on the concave pinna (**b**), eyelids (**c**), and lips and gums (**d**,** e**,** f**,** g**). Photo courtesy of Drs. Natalie Gedon (**a**, **b**), Monika Linek (**c**), Thierry Olivry (**d**, **e**, **f**, **g**)

The information about the clinical aspect of canine MMP is based on 54 published cases for which a detailed description is available [7, 20, 21, 25–27]. Primary lesions such as vesicles and/or bullae are not always captured and, when present, they are transient with a rapid progression to deep erosions and/or ulcers (Fig. 5). Lesions are usually distributed in a bilaterally symmetric pattern. Scarring is reported infrequently in dogs (10; 19%), which could be either due to lack of attention to this clinical feature, or, potentially, due to a variation in disease presentation as seen in humans. For example, the oral cavity, the most commonly affected body area in canine MMP (34; 63%), is infrequently accompanied by any obvious scarring in human MMP [23]. Within the oral cavity, gingivae (31; 57%) and hard and/or soft palate (24; 44%) are frequently affected, while tongue lesions are detected less often (9; 17%). Other commonly affected areas in dogs include labial and perilabial (29; 54%), nasal planum/perinasal (32; 59%), eyelids/periocular (24; 44%) and perianal/perigenital areas (25; 46%) and concave pinnae (19; 35%) (Fig. 5). The ocular mucosa (i.e., conjunctiva, sclera) and the palpebrae are rarely affected in dogs (2/54; 4%), while nasopharyngeal, laryngeal, and oesophageal involvement, which, in people, are all high-risk phenotypes frequently associated with a loss-of-function due to chronic scarring [28], have not been reported in dogs yet. Lesions affecting haired skin are infrequent (10; 19%), usually involve pressure points and high friction areas (e.g., scrotum, elbow, groin, axillae, interdigital, periungual) and are of minor severity. This is similar to humans, where skin involvement is infrequent (3/30; 10%) [29].

Non-dermatological signs such as lethargy, pain, halitosis and ptyalism are reported in about half of the canine cases, and they are usually dependent on the severity of mucosal and skin lesions.

#### Histopathology

**Fig. 6:**
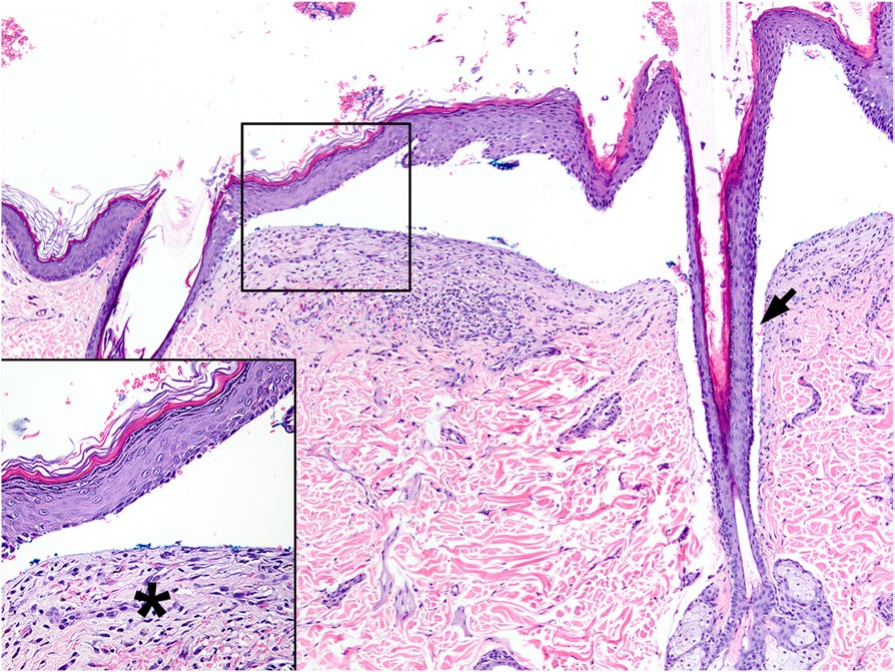
Histopathology of canine mucous membrane pemphigoid: A large subepidermal cleft, devoid of inflammatory cells, is present that spans a hair follicle, involves a hair follicle infundibulum (arrow), and is bordered by fibrosis below (box insert, asterisk). Haematoxylin and eosin

Histopathological features of canine MMP are similar to those reported in people [23]. Subepidermal or submucosal vesicles, which are commonly ruptured, are often devoid of inflammation (Fig. 6) [26], but can contain a few neutrophils, eosinophils, scant fibrin and/or a few erythrocytes. Vesicles are very small to large, sometimes span several follicles, and occasionally extend down hair follicle infundibula (Fig. 6). Scattered individual apoptotic keratinocytes can be seen in the basal and/or suprabasal epidermis or mucosa but are usually rare or are only a few. Dermal inflammation is variable and ranges from non-inflammatory lesions to more typical perivascular infiltrates of mild to moderate neutrophils (73%) mixed with lymphocytes and plasma cells that are often accompanied by eosinophils (55%) [26]. Rowing of individual neutrophils and/or histiocytes just below the BMZ occasionally occurs but is less common than in canine epidermolysis bullosa acquisita [26, 30]. Subepidermal microvacuoles can range from subtle to prominent, but are uncommon. In most cases, but not all, superficial dermal fibrosis (Fig. 6) occurs below vesicles or intact epithelium and is reorganizing or appears as a thin band of hyperplastic resident fibrovascular stroma, mimicking granulation tissue. A PAS stain (and/or collagen IV immunohistochemistry (IHC) of biopsy sections, if available), can be performed. Positive staining on the dermal side (floor) of the blister is expected, which can be used to differentiate MMP from epidermolysis bullosa acquisita (EBA) (Fig. 4). Indeed, EBA phenotypically mimicking MMP (aka. MMP-like EBA) that is recognised in humans could be differentiated from a true MMP either by an antigen-specific ELISA or by identification of the level of the split by visualizing the position of the lamina densa by PAS or collagen IV IHC [31]. Unfortunately, sensitivity of these latter tests, especially of PAS staining, is limited due to the BMZ degradation during the blister formation [18].

#### Immunopathology

Direct IF uncovered skin or mucosal-bound autoantibodies, particularly IgG (52/56; 93%), deposited along the BMZ in most dogs affected with MMP [6, 7, 20, 21, 25, 26, 32]. These results appear to be higher compared to humans with a single biopsy assessment (69%) [15], which could be explained by the fact that a positive direct and/or indirect IF results were required to confirm the diagnosis of MMP in dogs in publications used for this analysis. In contrast, because of the low sensitivity of the direct and indirect IF in human MMP, the detection of tissue-bound or circulating anti-BMZ antibodies is not a required diagnostic criterion in people with clinically and histologically compatible signs [15]. Additionally, direct IF also uncovered BMZ-bound complement C3 (33/44; 75%), IgM (19/40; 48%) and IgA (13/30; 43%) in affected dogs [7, 20, 25, 26, 32].

The use of a salt-split buccal mucosa substrate for indirect IF increases the sensitivity to detect circulating anti-BMZ autoantibodies in AISBDs, including MMP [33]. Nonetheless, circulating anti-BMZ IgG could only be detected in 33 of 43 dogs (77%) [7, 25, 26]. This lower detection rate is not unique to canine MMP; a low prevalence of circulating anti-BMZ IgG was also reported in people, especially when lesions were confined to the oral cavity [15, 23]. Circulating anti-BMZ IgE and IgA were detected in 8 of 15 dogs (53%) and 2 of 27 dogs (7%), respectively [33]. Detection of circulating anti-BMZ IgM antibodies has not been reported in dogs with MMP [7, 25, 26].

Like the human disease, canine MMP has been shown to be immunologically heterogeneous with autoantibodies targeting proteins of the basement membrane such as NC16A domain of collagen XVII (BP180), a major autoantigen, BPAG1e (BP230) or laminin-332 [6, 7, 25]. Because of this heterogeneity in target antigens, indirect IF testing on salt-split tissue can lead to three distinct fluorescence patterns for this disease: circulating anti-BMZ IgG autoantibodies can bind to the epidermal (most cases), dermal or both sides of salt-induced splits (Fig. 3). Autoreactivity against other basement membrane proteins described in some affected humans, such as the α6β4 integrin, laminin-311 or collagen VII, has not been confirmed in dogs yet.

#### Treatment and outcome

As in humans, canine MMP appears to follow a chronic evolution with a waxing-and-waning course with lesions often recurring at the same locations, which, in cases with a long-lasting disease, is the likely cause for the scarring. The treatment and outcome information summarised below was obtained from 25 MMP-affected dogs reported previously. A spontaneous remission was not reported in any of these dogs, but various treatment regimens were able to induce complete remission in most patients. Drugs frequently used included oral prednisone/lone (0.5–4 mg/kg/day), tetracycline (250 or 500 mg three times daily depending on dog’s size) or doxycycline (5–10 mg/kg once or twice daily) and niacinamide (250 or 500 mg twice or three times daily), ciclosporin (5–10 mg/kg/day), azathioprine (1.5–2.4 mg/kg/day), chlorambucil (0.1–0.2 mg/kg/day), mycophenolate mofetil (20 mg/kg twice daily) or dapsone (1.2 mg/kg twice daily) [20, 21, 25–27]. These drugs were used as a monotherapy or in various combinations. Tetracycline antibiotics and niacinamide alone or in combination with another immunosuppressant was the most common drug combination that induced disease remission, while monotherapy with glucocorticoids appeared to be the least successful to help with disease control [25–27]. The median time to complete remission of canine MMP with treatment was 33 weeks and ranged from 6 to 64 weeks [26]. Frequent disease flares were reported by most authors and were often associated with a drug dosage reduction or discontinuation; in some dogs, however, the cause for the flare-up was not identified.

#### Summary

**Fig. 7:**
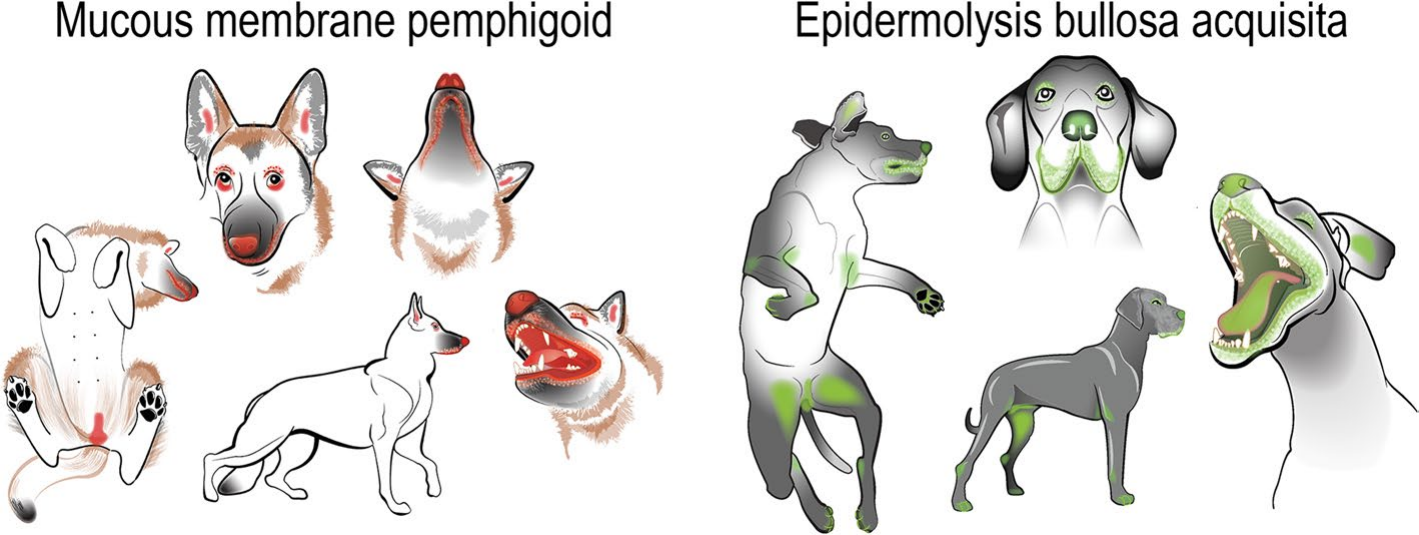
Canine mucous membrane pemphigoid and epidermolysis bullosa acquisita - Lesion distribution diagram. Artist: Alice Harvey

Canine MMP is a naturally occurring, chronic and recurrent AISBD that preferentially affects mucosae and mucocutaneous junctions (Fig. 7). It is the most common AISBD in dogs and German shepherd dogs are an over-represented breed.

Because of its immunological heterogeneity and lack of specialised laboratory testing available, the identification of the target basement membrane antigen is currently not required for diagnosis confirmation. Like in human MMP, we proposed that the diagnosis of MMP in dogs should be based on clinical signs (an adult onset blistering disease involving predominantly mucosae and mucocutaneous junctions) and the histological confirmation of dermo-epidermal separation (Fig. 6). A positive PAS staining and/or anti-collagen IV IHC should label the dermal side (floor) of the blister in biopsies; however, this result does not help to differentiate MMP from most AISBDs but should help distinguish MMP from EBA.

Although most dogs will reach complete remission of the disease with treatment, frequent flare-ups may be observed.

### Mucous membrane pemphigoid in other animal species

A naturally occurring MMP has been described in four cats (one of the cats being originally published as BP [case #1 in the original publication] before the consensus renaming of human MMP in 2002) [10, 13, 24]. The age of onset varies from one to 7 years and all four cats exhibited mucosa/mucocutaneous junction-dominant blistering skin disease with vesicles and/or erosions and ulcers on the lips (4/4; 100%) and in the oral cavity (3/4; 75%), including palate (3/4; 75%), gingivae (2/4; 50%) and tongue (1/4; 25%) (Fig. 5e-g). Additionally, the nasal planum (2/4; 50%), conjunctivae (2/4; 50%), eyelids (1/4; 25%), and concave pinnae (2/4; 50%) can also be affected. In these cats, histopathology revealed dermo-epidermal separation with none-to-minimal dermal inflammation composed of dendritic/histiocytic cells and occasional neutrophils and eosinophils [10, 13, 24]. Two of the four cats exhibited, at the time of active skin lesions, unexplained very high elevations of the alanine aminotransferase enzyme; in one case where it was followed, the activity of the enzyme abated with the successful MMP treatment [24]. Immunotesting was performed in two of the four cats and revealed autoantibodies targeting collagen XVII in one cat and laminin-332 in the other cat [10, 13]. Two of the cats responded to oral glucocorticoid monotherapy; the type and dosage (prednisone 2 mg/kg twice daily) were reported for only one of these cases [10, 13]. The other two cats were successfully treated with dexamethasone (0.1–0.2 mg/kg/day) and doxycycline (5 mg/kg twice daily) [24]. Attempts to discontinue all medications resulted in rapid flare-ups in three of the four cats (75%) and, therefore, an intermittent administration of glucocorticoids at lower dosages were necessary. A long-term remission off drugs (6 months) was reported in one cat (25%) [10].

### Epidermolysis bullosa acquisita

Epidermolysis bullosa acquisita, a deep AISBD with autoantibodies against collagen VII, has been recognised in people and dogs. In both species, it represents a severe blistering disease affecting haired skin, mucosae and mucocutaneous junctions. Relevant information on canine EBA was extracted from 23 previously published cases and from one additional unpublished case with supportive immunological test results [4, 30, 34, 35].

### Canine epidermolysis bullosa acquisita

#### Prevalence and signalment

Epidermolysis bullosa acquisita is the second most common AISBD of dogs (26% of all AISBDs) [2]. Most affected dogs are young (median: 1.2 years; range: 4 months – 8 years) males (M:F ratio = 2.3), with almost half of them (45%) developing lesions before 1 year of age [30]. A childhood EBA has also been recognised in people; however, this disease usually affects adults, mostly in the fourth to fifth decade of their lives [36, 37]. Great Danes account for more than half (14/24; 58%) of the reported dogs, and German shorthaired pointers are the second most commonly reported breed (3/24; 13%) [4, 30, 34, 35]. That young adult dogs from a single breed develop EBA suggests a strong genetic predisposition for this disease.

#### Clinical signs

**Fig. 8:**
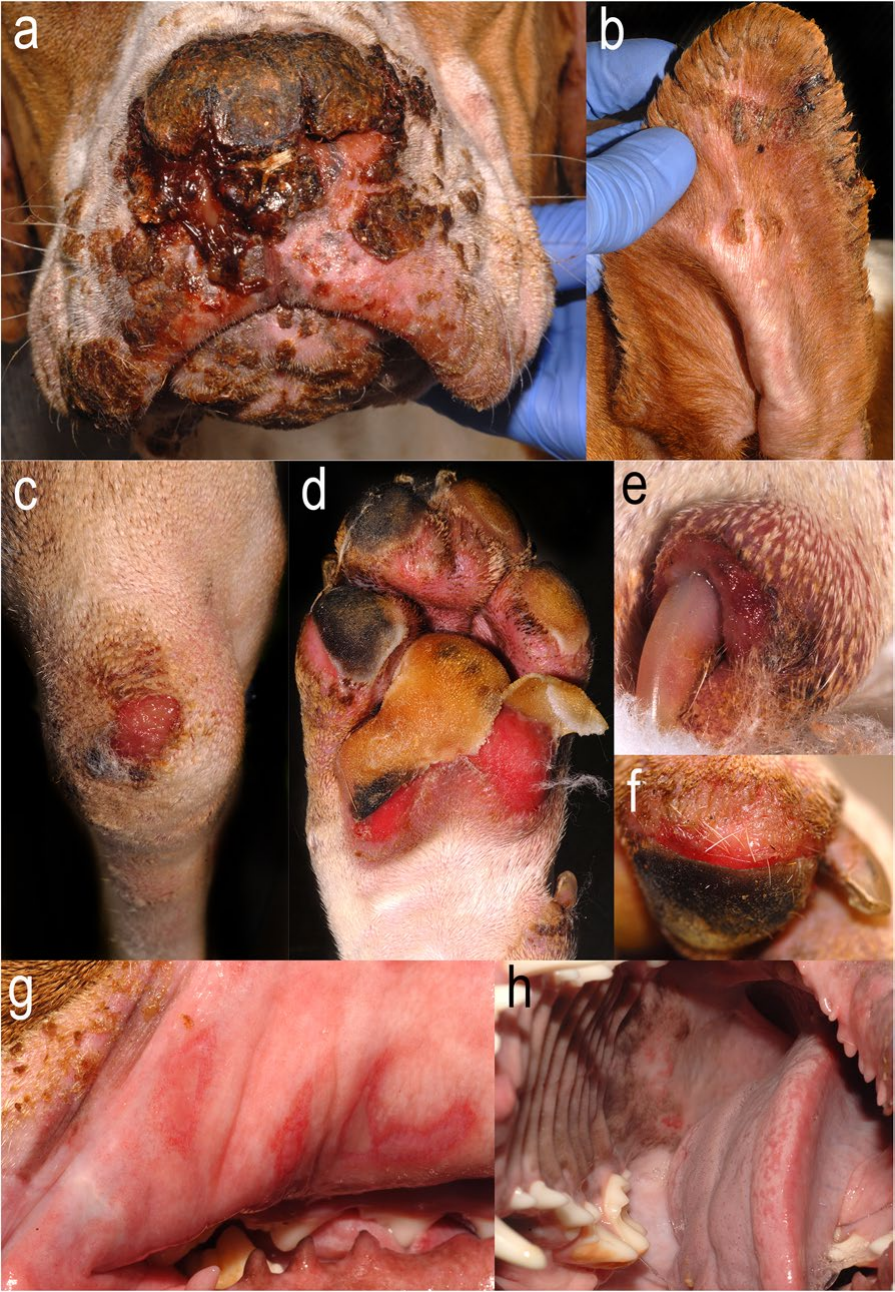
Canine epidermolysis bullosa acquisita: Haired skin involvement is common and often composed of only secondary lesions such as erosions, ulcers and crusts, which in this dog affect the nasal planum (**a**), concave pinna (**b**), pressure point areas such as elbows (**c**), and periungual (**e**). Footpad sloughing is common (**d**, **f**). Mucosal involvement is also frequently encountered; herein, deep erosions or ulcers affect the buccal mucosa of lips and tongue (**g**, **h**)

The current knowledge about the clinical aspect of canine EBA was extrapolated from 23 published and one unpublished case [4, 30, 34, 35]. The characteristic skin lesions seen in dogs with EBA are tense vesicles and bullae (22/24; 92%) progressing into deep erosions and ulcers (24; 100%). Additional lesions include erythematous macules and patches (18; 75%) or papules and wheals (10; 42%), all of which could precede the development of blisters at the same site. Epidermolysis bullosa acquisita is a blistering disease that affects both mucosae, mucocutaneous junctions and haired skin (Fig. 7). The oral cavity (22; 92%), lips (19; 79%), concave pinnae (19; 79%) and haired skin in areas of friction and pressure (e.g., groin, axillae, pressure points) are involved most frequently (22; 92%) (Fig. 8). In contrast to most dogs with MMP, dogs with EBA often exhibit footpad sloughing (17; 71%) (Fig. 8d, f). Pruritus and pain are frequently observed (38 and 86% of affected dogs, respectively), and systemic signs such as fever, lethargy, lymphadenopathy and anorexia are seen in almost all cases. An exception was a single dog with a very mild disease in which only several small lesions were scattered across the trunk and head [34]. This presentation resembled the Brunsting-Perry-like phenotype, which is one of the non-classic/non-mechanobullous forms of EBA described in people and characterised by blisters confined to the head and neck, minimal inflammation, and tendency to scar [14]. Additional non-classic/non-mechanobullous forms of EBA in people include BP-like (inflammatory blisters in pressure, friction, or trauma-prone areas; mucosal involvement is possible), MMP-like (usually inflammatory blisters on the mucosae and mucocutaneous junctions) and linear IgA-like EBA (exclusively IgA deposit along the BMZ). As apparent from their names, these forms of EBA resemble other AISBDs. The last form described in people is the non-inflammatory classic/mechanobullous EBA, which presents with skin fragility, blisters, and deep erosions on a non-inflamed skin, particularly in areas of pressure or friction such as dorsal hands, elbows, knees, Achilles tendons, and feet [14]. If we were to apply the human EBA phenotypic classification to the canine disease, most dogs with EBA would fit the so-called BP-like EBA variant [14]. The other two inflammatory variants, MMP-like and linear IgA-like EBA, and the non-inflammatory classic/mechanobullous EBA variant have not been described in dogs yet.

#### Histopathology

**Fig. 9:**
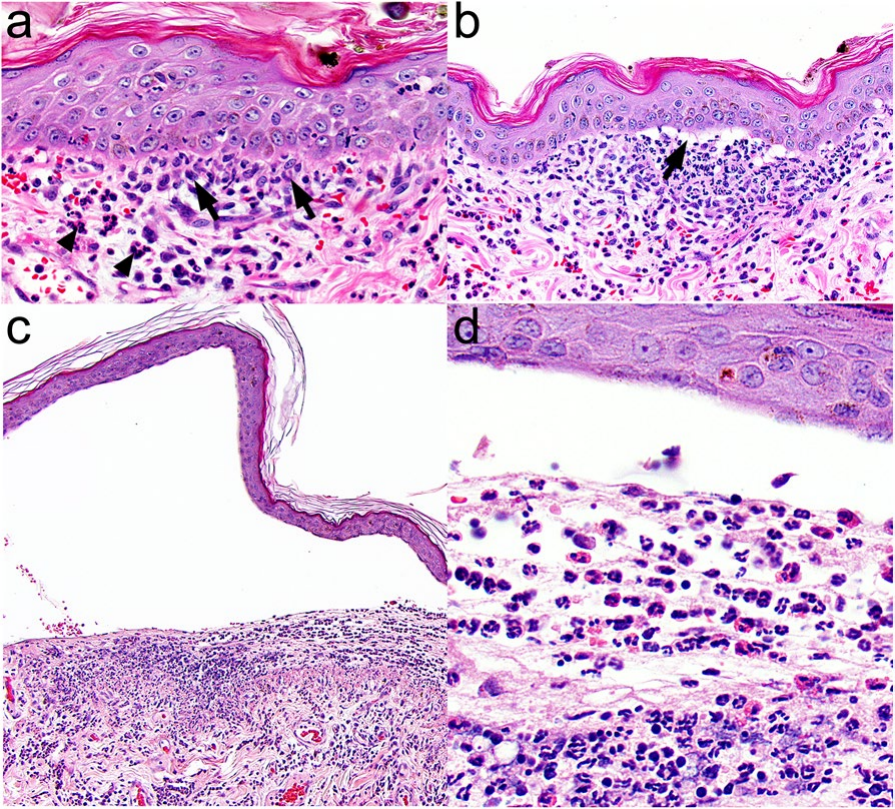
Histopathology of canine epidermolysis bullosa acquisita: **a** Neutrophils and macrophages form a row (arrows) along the basement membrane zone (BMZ) in the absence of a subepidermal cleft. Such alignment is not observed with eosinophils (arrow heads). **b** Neutrophils and fewer macrophages cluster below the BMZ and are associated with a microscopic subepidermal cleft that appears to form as clusters of small, subepidermal, clear vacuoles (arrow). **c** A large subepidermal vesicle contains neutrophils, eosinophils, and a small amount of fibrin in the lumen over similar inflammation in the superficial dermis. **d** A higher magnification image of the vesicle in image “c” showing many eosinophils mixed with neutrophils in the vesicle lumen. Haematoxylin and eosin

**Fig. 10:**
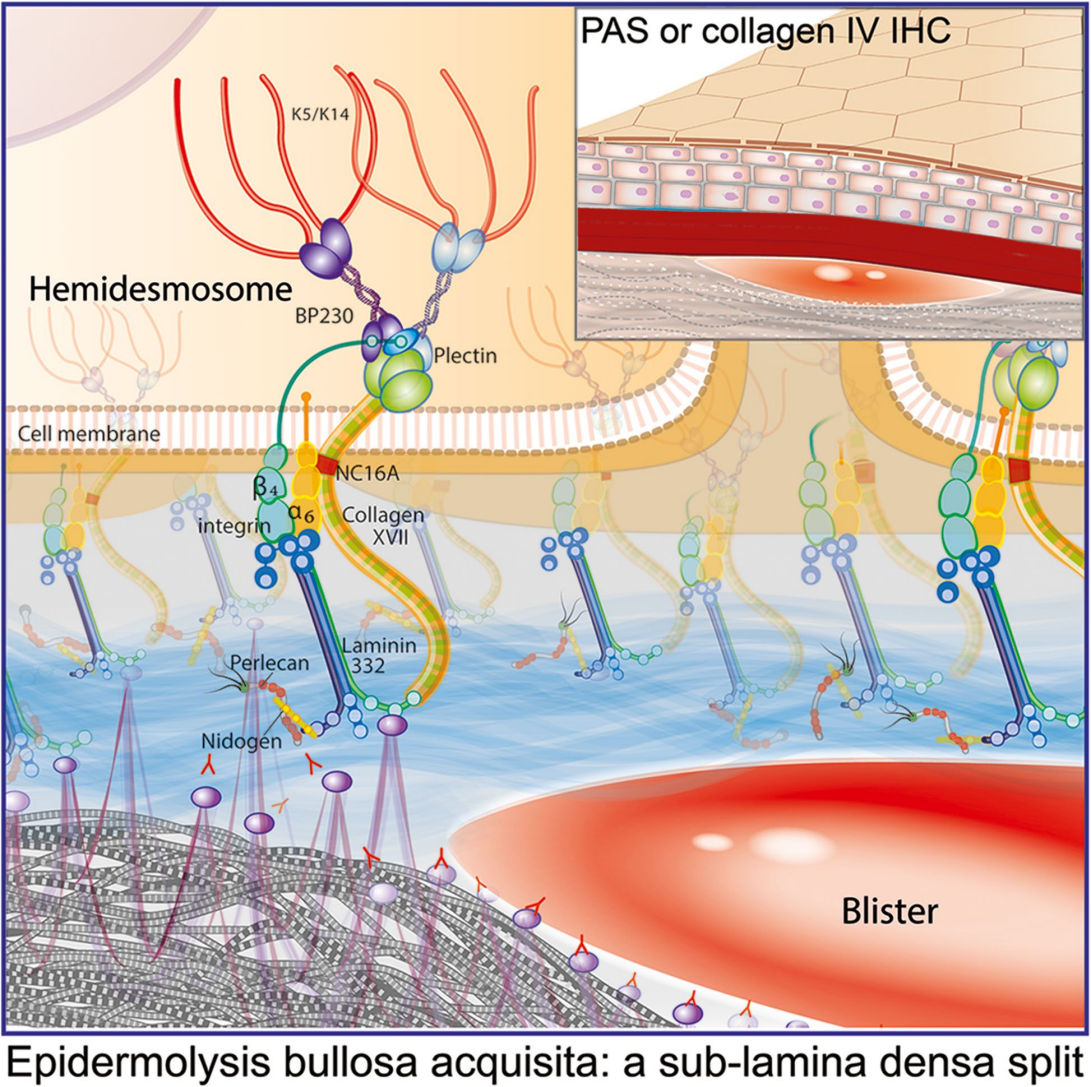
Anti-collagen VII autoantibodies and a sub-lamina densa split in epidermolysis bullosa acquisita: A PAS or collagen IV immunohistochemical staining in this case will be on the roof of the biopsied blister (right upper corner insert). Artist: Alice Harvey

Detailed histopathological description can be found for 17 dogs with EBA [30]. Like in other AISBDs, dermo-epidermal separation leading to vesicle formation is a typical finding in EBA (Fig. 9). Vesicles are devoid of any inflammation (76%) or contained variable numbers of neutrophils (94%), which in some dogs are mixed with eosinophils (41%). A small amount of fibrin and/or minor haemorrhage is often found in some vesicles. Vesicles range from microscopic to very large and sometimes involve hair follicle infundibula. The clefted epidermis undergoes degenerative changes, including occasional apoptosis or coagulation necrosis, and basal predominant atrophy. Subepidermal vacuolation as well as rowing of neutrophils and/or histiocytes occurr just below the BMZ (Fig. 9), sometimes with subepidermal clustering. A thin band of granulation occurrs in the superficial dermis below vesicles, or intact epidermis, in a minority of cases. Superficial dermal, perivascular to interstitial, inflammation containes neutrophils (100%), as well as lymphocytes and plasma cells, and a variable number of eosinophils (71%) (Fig. 9). Eosinophils may, occasionally, outnumber neutrophils. Due to the presence of eosinophils in several AISBDs and the variable presence of eosinophils in EBA, the lack of eosinophils cannot be used to differentiate EBA from other AISBDs. Likewise, the type of inflammation, particularly the lack of eosinophils, showed insufficient specificity (68%) to reliably distinguish EBA from BP in people [18]. Because of the sub-lamina densa split in EBA (Fig. 10), positive collagen IV IHC staining on the epidermal side (roof) of the blister could be used as a valuable diagnostic tool to differentiate this entity from other AISBDs (Figs. 4 and 10 insert). A collagen IV IHC on formalin-fixed paraffin-embedded sections from dogs with EBA showed relatively high positive and negative predictive values (83%), but a low sensitivity (71%) [38]. Similarly, positive PAS staining on the epidermal side (roof) of the blister could be used to support the presumptive diagnosis; however, the sensitivity of this test is also limited, likely due to BMZ degradation during the blister formation [18]. Indeed, despite the high specificity (95%), the sensitivity of this test in human EBA in a recent study by Gardner was only 25% [18].

#### Immunopathology

In most dogs affected with EBA, tissue-bound autoantibodies, predominantly IgG (17/20; 85%), were detected along the basement membrane zone using direct IF. In the serum, circulating anti-BMZ IgG were detected by indirect IF using salt-split canine buccal mucosa tissue in most dogs (22/24; 92%). With this method, autoantibodies were found to bind to the dermal side (floor) of the salt-split blister (Fig. 3). Using immunoblotting, ELISA with human recombinant proteins, or canine NC1-transfected 293 T cells, autoantibodies targeting the NC1 domain of collagen VII were confirmed in the majority of tested dogs (22/23; 96%); rare dogs also had autoantibodies against the shed NC2 segment (4/12; 33%) [4, 30, 34, 35]. By immunogold transmission electron microscopy, the IgG antibodies from the first described patient were visualised to bind to the end of anchoring fibrils in the superficial dermis [4]. Altogether, these results suggest that canine EBA is a remarkably close immunological homologue of the human disease.

#### Treatment and outcome

Information about the treatment and outcome of canine EBA is based on data from 24 dogs (23 published and the one unpublished) [4, 30, 34, 35]. A spontaneous remission of signs was not reported in any of these dogs. Six dogs were euthanised due to a lack of response to treatment, whereas one dog was euthanised immediately after the diagnosis was confirmed; most of these cases were in the early stages of recognition of this disease when the most suitable therapy was not identified. In the remaining 17 dogs, a complete disease remission was obtained within the first 2–3 months using various treatment regimens. In most of these cases, the full remission was achieved with a combination therapy with oral glucocorticoids (prednisone dosage equivalent of 2–4 mg/kg/day) and various non-steroidal immunosuppressants (azathioprine, mycophenolate mofetil, etc.) at standard immunosuppressive dosages. Colchicine (0.03 mg/kg/day), a drug frequently used in human EBA, was used concurrently with other immunosuppressive drugs in about one third of successfully treated dogs, suggesting an added benefit in this disease. Other, anecdotal treatments found in the literature in conjunction with immunosuppressive therapy included intravenous immunoglobulins and doxycycline. In almost half of the dogs (8/17; 47%), immunosuppressive drugs could be discontinued without further recurrence of signs.

#### Summary

Canine EBA is a naturally occurring AISBD with collagen VII autoreactivity affecting mucosae, mucocutaneous junctions and haired skin. Footpad sloughing is frequent (Figs. 7 and 8). Systemic signs such as fever, lethargy, anorexia and pain are common.

Because of the lack of advanced immunotesting in veterinary dermatology, the diagnosis of EBA is currently based on suggestive clinical signs (a severe acquired blistering disease affecting haired skin, mucosae, and, frequently, footpads, a younger age of onset, and a predisposition towards male Great Danes), histological confirmation of a dermo-epidermal separation with neutrophil-rich inflammation and demonstration of the blister below lamina densa using PAS and/or collagen IV IHC staining. The latter, due to its low sensitivity, is not always achievable. If available, indirect immunofluorescence using salt-split buccal mucosa tissue can be used to demonstrate anti-BMZ IgG (+/− other Ig classes) along the dermal side (floor) of the artificial salt-split blister, and thus help to distinguish it from BP or other AISBDs with anti-collagen XVII or anti-BPAG1e autoimmunity. This staining will not distinguish EBA from other AISBDs with anti-laminin 332 autoimmunity, however (Figs. 3 and 4).

Most dogs with EBA can be successfully controlled with a combination therapy composed of a glucocorticoid and non-steroidal immunosuppressant within a few months. Colchicine, because of its anti-neutrophilic properties [39], is often used concurrently, but the evidence for its benefit to help manage canine EBA is still limited. Once in remission, the dosages of used medications can be markedly reduced or discontinued in most dogs.

### Epidermolysis bullosa acquisita in other animal species

A naturally occurring EBA has not been reported in any animal species other than dogs.

## Bullous pemphigoid

Bullous pemphigoid is the most common AISBD in people, thus explaining why most dogs with blistering and/or erosive skin lesions were historically given this diagnosis [32, 40–45]. However, in contrast to people, canine BP is seen rarely. Naturally occurring BP has also been described in cats, horses, pigs and possibly a macaque. In all species except horses, BP is a haired skin-predominant AISBD with none or mild mucosal involvement, and it is usually not associated with overt systemic signs. Relevant information on animal BP for this review has been extracted from the published literature and cases submitted to the NCSU immunodiagnostic laboratory. Altogether, we included data from nine dogs [22, 46], two cats [10, 19], seven horses [11, 47–51], a dozen Yucatan minipigs [12], and one rhesus macaque [52].

### Canine bullous pemphigoid

#### Prevalence and signalment

Bullous pemphigoid is rarely diagnosed in dogs (10% of all AISBDs), but it is still the third most common AISBD in this species [2]. Among described dogs, males are affected twice as often as females (a M:F ratio 2). A breed predisposition could not be determined due to the low number of cases, but the two breeds with more than one reported case were the dachshund (2/9; 22%) and German shepherd dog (2/9; 22%). The median age of onset was 5 years (range: 10 months – 15 years).

#### Clinical signs

**Fig. 11:**
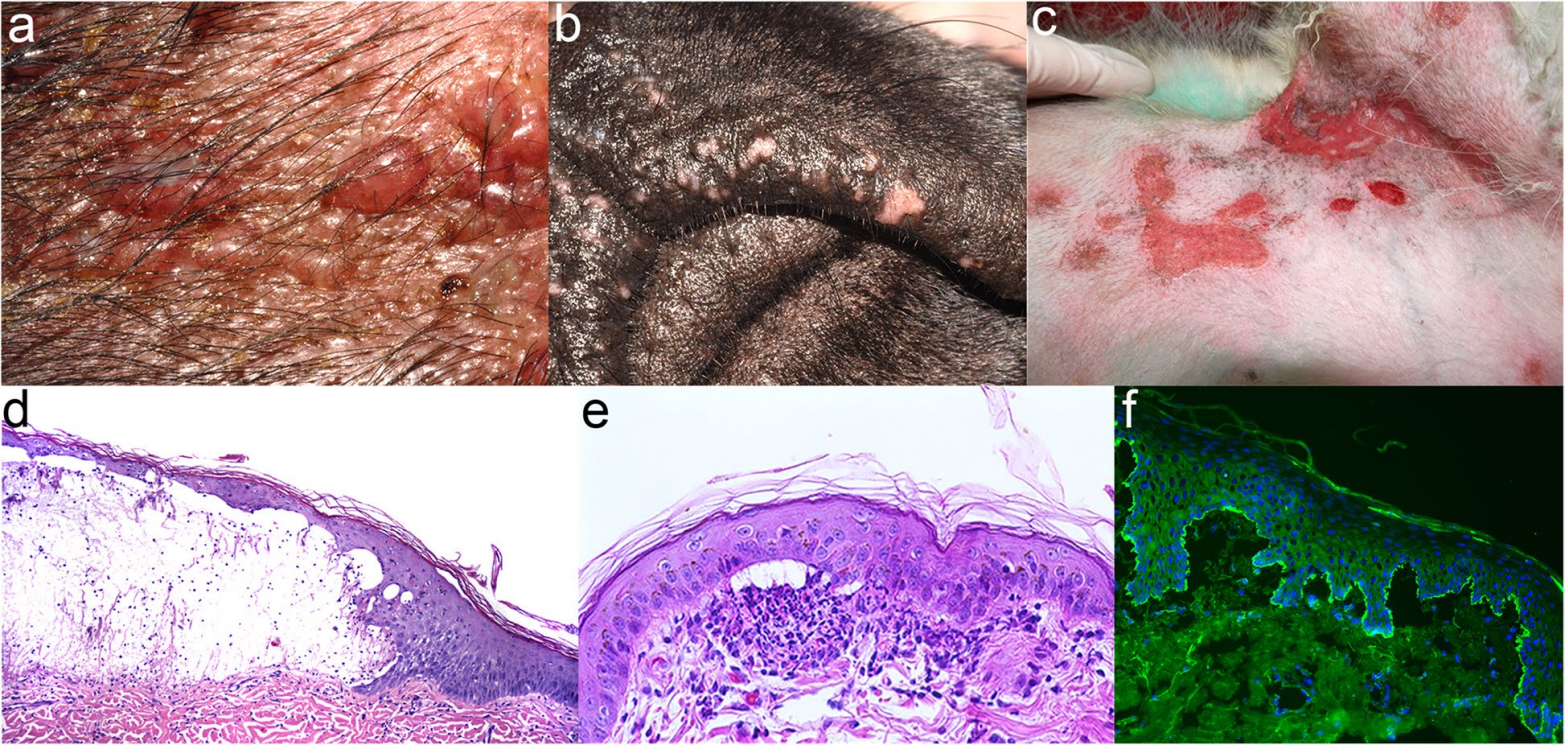
Canine bullous pemphigoid: Vesicles and deep erosions affecting predominantly the haired skin (**a**, **c**) with milder mucosal and mucocutaneous junction involvement (**b**). Histopathology is characterised by dermoepidermal separation (**d**, **e**) with inflammation that often contains numerous eosinophils (**e**). Indirect immunofluorescence on a salt-split buccal mucosa demonstrated IgG along the roof of the blister (**f**). Photo courtesy of Drs. Thierry Olivry (**a**, **b**, **d**-**f**) and Ekaterina Kuznetsova (**c**)

The information about the clinical aspect of canine BP is based on nine cases [22, 46]. In these dogs, the most common skin lesions were deep erosions/ulcers (8/9; 89%) followed by erythematous macules, patches or plaques (6; 67%), and crusts (6; 67%). Intact vesicles or bullae (4; 44%) were captured less frequently (Fig. 11a). Dogs exhibited lesions on haired skin (9/9; 100%) as well as mucosae (6; 67%) and mucocutaneous junctions (6; 67%) (Fig. 11b). Haired skin involvement was most common in friction/pressure point areas such as axillae, groin and elbows (5; 56%) and on the pinnae (5; 56%) (Fig. 11c). Footpads, claw fold and perianal/perigenital areas were affected infrequently (2 each; 22%). In general, lesions affecting mucosae and mucocutaneous junctions were of milder severity than those of skin and involved lips (5; 56%) (Fig. 11b), tongue (3; 33%), gingivae (2; 22%), nasal planum (2; 22%), eyelids (1; 11%; not conjunctivae) and palate (1; 11%).

Dogs affected with BP were otherwise healthy, and systemic signs were usually not observed.

#### Histopathology

Like in other AISBDs, dermo-epidermal separation leading to vesicle formation is a typical finding in BP [46]. Formed vesicles can be either devoid of inflammation (7/8; 88%) or contain variable numbers of neutrophils (5/8; 63%) and/or eosinophils (4/7; 57%) (Fig. 11d-e). Similar inflammatory infiltrate can be detected in the upper dermis. Eosinophils occur in other AISBDs in dogs and do not help to differentiate BP. In some dogs, numerous degranulated mast cells are depicted in the upper dermis [22, 46]. A positive PAS stain on the dermal side (floor) of the blister could help to differentiate BP from EBA but not MMP; however, the sensitivity of this test is limited, likely due to the BMZ degradation by the concurrent inflammation. Similar staining pattern would be expected with anti-collagen IV antibody, though data to support this statement are limited to only a single case [38].

#### Immunopathology

Biopsies from four (91%) and two (40%) of the five tested dogs with BP possessed tissue-bound IgG and IgM autoantibodies, respectively, deposited along the BMZ. Tissue-bound complement (C3) was detected in one of the five tested dogs (20%) (Olivry: unpublished data). Circulating IgG, predominantly IgG1, could be detected using salt-split canine buccal mucosa tissue in all tested dogs with BP ([33] and Olivry: unpublished data), where they bound to the epidermal (roof) side of the blister in the salt-split tissue (Fig. 11f). Circulating IgE targeting BMZ, which have been under scrutiny lately in human BP for their contribution to pathogenicity, were detected in 60% of tested dogs. Autoantibodies (IgG) were shown to target collagen XVII (BP180 antigen) in most dogs, while BPAG1e (BP230) was shown to be a minor antigen [3, 46, 53]. Using synthetic overlapping peptides spanning the human NC16A domain of collagen XVII, IgG from dogs with BP were found to recognize mostly the carboxy-terminal end of this segment, as in humans with BP [46].

#### Treatment and outcome

Information about treatment and outcome is limited to seven dogs with BP. A spontaneous remission was reported in one dog. In the six treated dogs, various treatment regimens induced complete remission of signs, including monotherapy with glucocorticoids (prednisone/lone 2 mg/kg/day; 3 dogs) or oclacitinib (1 mg/kg/day; 1 dog), or combination therapies with prednisone/lone (2 mg/kg/day), azathioprine (2 mg/kg/day) and pentoxifylline (1 dog), and doxycycline (10 mg/kg/day) and niacinamide with occasional prednisone (1 dog). In 40% of these dogs, medications were discontinued without any apparent recurrence of the disease.

#### Summary

Canine BP is a naturally occurring AISBD with collagen XVII autoreactivity affecting predominantly friction and pressure point areas of haired skin (Fig. 1a-c). Mucosal and mucocutaneous junction lesions are reported in the majority of dogs with BP, but they are usually milder in its severity compared to other AISBDs like MMP and EBA. This varies from human BP where mucosal involvement is seen in only a small proportion of patients (10–35%) [54]. Footpad sloughing, in contrast to EBA, is only a rare feature of BP. Systemic signs are rarely observed.

Because of the lack of advanced immunotesting in veterinary dermatology, the diagnosis of BP is currently based on suggestive clinical signs (haired skin predominant, acquired blistering skin disease without or with milder mucosal involvement) and histological confirmation of a dermo-epidermal separation, often rich in eosinophils (Fig. 11d-e). Unfortunately, due to the presence of eosinophils in other AISBDs, the presence of eosinophils cannot be used to differentiate BP from other AISBDs. If available, a positive PAS and/or anti-collagen IV immunostaining on the dermal side (floor) of the blistered skin could further support the diagnosis of BP and distinguish it from the more common EBA. The sensitivity of these tests is, however, limited [18, 38]. If available, indirect immunofluorescence using salt-split buccal mucosa tissue can be used to demonstrate anti-BMZ IgG (+/− other classes) along the epidermal (roof) side of the artificial salt-split blister, and thus help to distinguish it from AISBDs with anti-laminin-332 and anti-collagen VII autoimmunity.

Canine BP responds well to a standard immunosuppressive therapy, with a long-term remission and later discontinuation of treatment is achievable in most dogs.

### Bullous pemphigoid in other species

Rare cases of a naturally occurring BP have been described in cats, pigs, horses and, possibly, in a rhesus macaque. Immunological confirmation, including the identification of the target antigen, is limited to only a single feline case [10], a dozen Yucatan minipigs [12], and two horses [11]. Additional reports included animals with compatible clinical and histological features in which anti-BMZ autoantibodies were detected via direct or indirect IF (three horses and the rhesus macaque), but in which a target antigen was not investigated [47, 48, 51, 52], and animals (one cat and one horse) in which the diagnosis of BP was based solely on clinical and histological features [19, 50].

In cats, lesions of BP appear to be of minimal severity, with vesiculation and erosions occurring predominantly on the ears, trunk and/or extremities [10 (case 2), 19 (presumed)]. Mucosal involvement can be seen but appears to be mild. Like in people and dogs, the single BP-affected cat in which advanced immunotesting was performed had IgG against the NC16A domain of collagen XVII [10]. In both reported cats, signs responded to treatment with glucocorticoids, though complete remission was achieved in only one of them [19]. In this cat, a partial response to tetracycline (250 mg/cat three times daily) was also observed [19].

**Fig. 12:**
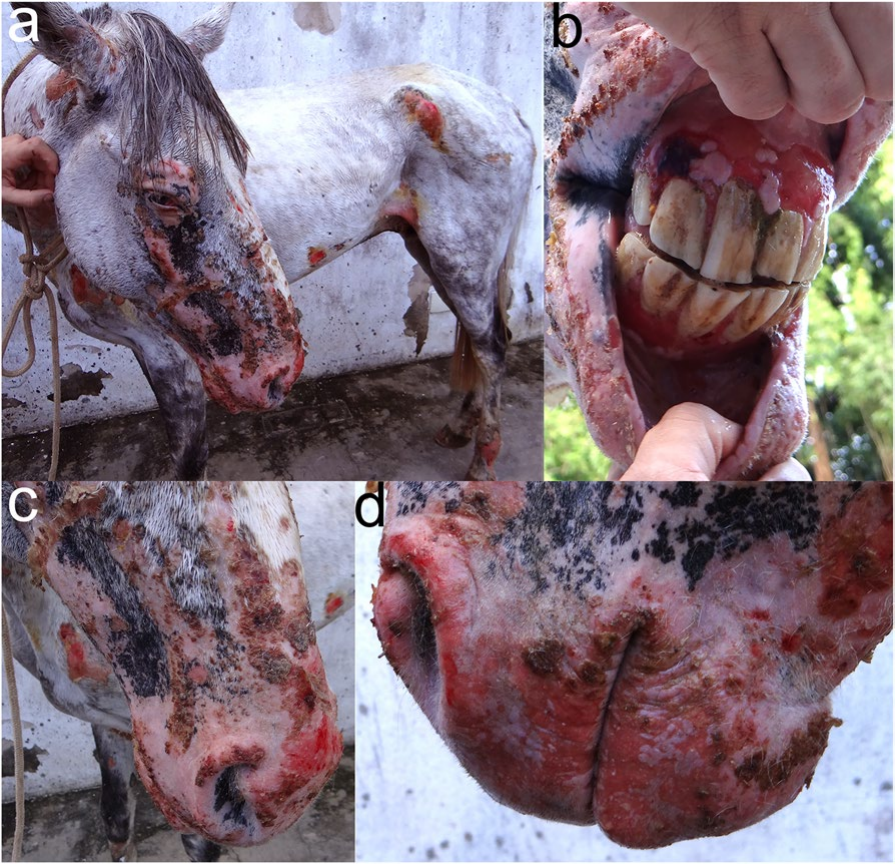
Equine bullous pemphigoid: Widespread deep erosions and ulcers affecting the haired skin (note the lesions on the pressure points, **a**), mucosae (**b**) as well as mucocutaneous junctions (**b-d**). Photo courtesy of Dr. Tiago da Cunha Peixoto (a case previously published by Fontes, et al) [50]

In most horses with BP, vesicles appeared suddenly and progressed rapidly into erosions and ulcers that became covered with crusts (Fig. 12). Lesions were usually widespread and affected both haired skin and mucosae, particularly the oral cavity (Fig. 12). Pruritus (2/4) or pain (2/4) was reported in all horses for which this information was available [47, 48, 50, 51]. Systemic signs such as lethargy, anorexia, weight loss, and dehydration were seen in most horses. In most reported cases, humane euthanasia was elected due to the disease severity, poor response to treatment or treatment-associated adverse events. Sera from horses with BP, in which further immunotesting was performed, contained IgG against the NC16A domain of collagen XVII [11].

**Fig. 13:**
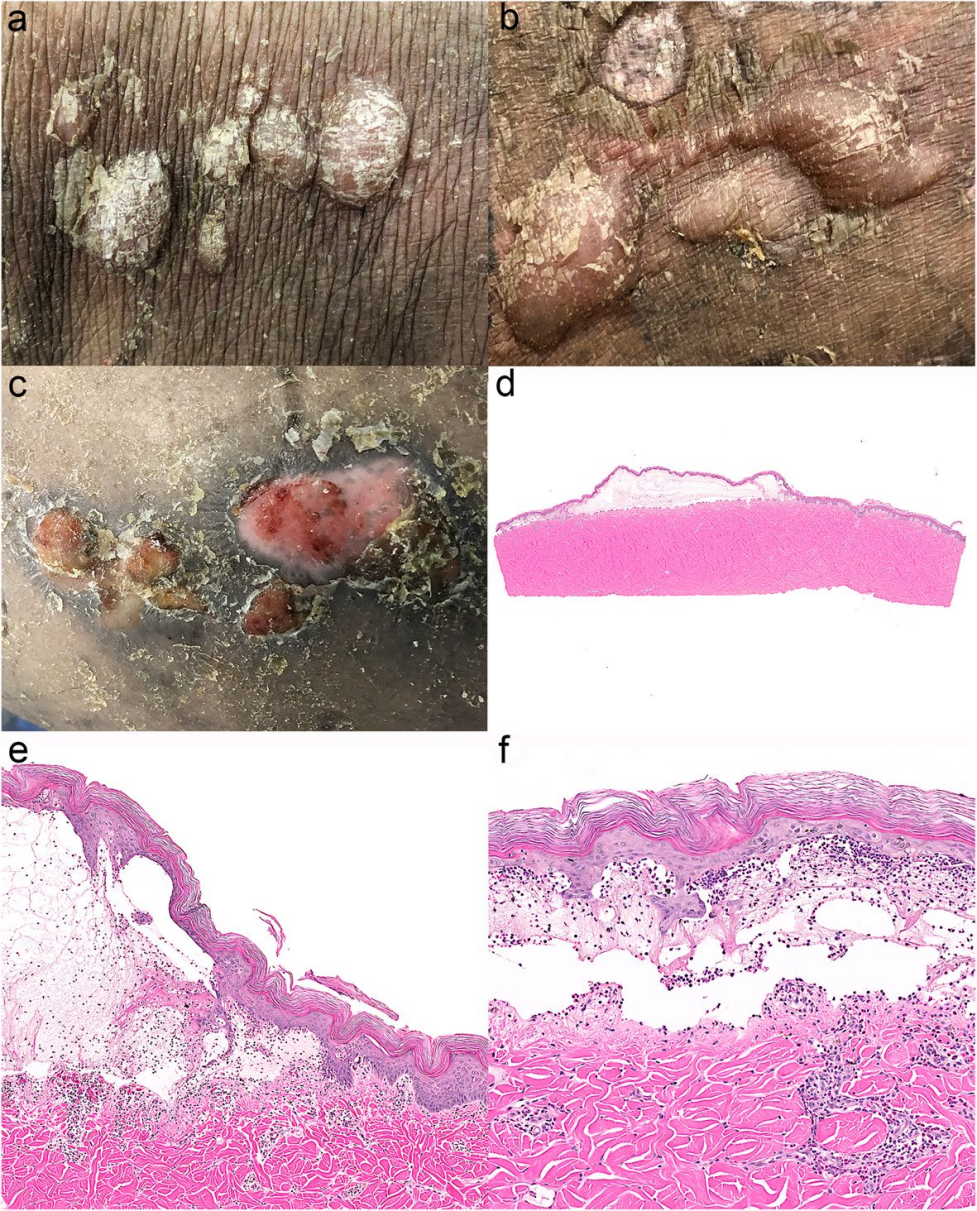
Miniature pig bullosa pemphigoid: **a** Vesicles and small bullae are present in a crop on the back with mild scaling. **b** Large bullae on the back are present next to a circular area of partial depigmentation that is bordered by a rim of hyperpigmentation and scaling, which is a site of a previously healed bulla. **c** Grouped erosions and shallow ulcers are sites of ruptured bullae and are well demarcated, circular, mildly crusted, and bordered by mild scaling and a rim of hyperpigmentation. **d** Low magnification histologic image of a bulla demonstrates subepidermal clefting. **e** and **f** Higher magnification images of the bulla in image “d” showing subepidermal clefting, eosinophils, neutrophils, and fibrin within the lumen of the bulla and a perivascular infiltrate of predominately eosinophils in the dermis. Haematoxylin and eosin

In young Yucatan minipigs from an experimental facility [55], clinicians observed trunk-dominant, clear to haemorrhagic tense vesicles progressing rapidly to erosions, and ulcers (Fig. 13a-c). In some pigs, erythema preceded vesicle formation. Mucosal involvement was usually not present. Histologically, lesions were indistinguishable from those of human BP and contained eosinophils and neutrophils in vesicles and bullae with similar perivascular inflammation in the dermis (Fig. 13d-f). Similar to other described species, sera from these pigs contained IgG against the NC16A domain of collagen XVII [12, 55]. In most of these pigs, lesions were reported to resolve with topical glucocorticoids. After a couple of active blistering episodes, there was no further recurrence of signs. In two pigs, immunization with human NC16 peptides led to a rise in BMZ IgG titres and local autoantibody deposition at the dermo-epidermal junction, but blistering could not be induced (Olivry: unpublished observations).

In the single case of *rhesus macaque* with presumed BP, tense and clear vesicles appeared on the nipples, shoulders and scalp of an animal undergoing experimental pancreatic transplantation. No mucosal lesions were reported. The dermo-epidermal separation was above the PAS-stained lamina densa, and direct immunofluorescence detected anti-BMZ IgG bound to the roof of the blister. The animal had a spontaneous resolution of clinical signs 2 weeks after the initial appearance of signs [52].

## Junctional epidermolysis bullosa acquisita (JEBA)

Junctional EBA is a rare AISBD that resembles canine EBA, but in which autoantibodies targeting laminin-332 are detected. Junctional EBA has been recognised in dogs [7]. A single human case with similar clinical and immunological features has been reported [56].

### Canine junctional epidermolysis bullosa acquisita

#### Epidemiology and signalment

In dogs, JEBA is a rare AISBD with only five described cases (6% of all AISBDs) [2]. Three dogs were Labrador or Chesapeake Bay retrievers, while the other two breeds were one Cairn terrier and one bearded collie. The median age of onset was 2.5 years (range: 6 months to 8 years), and there were three males and two females [7].

#### Clinical signs

All five dogs experienced an acute onset of vesicular and erosive skin lesions, that appeared and were distributed like those of EBA. Frequently affected areas were the concave pinnae (5; 100%), oral cavity (5; 100%), footpads (4; 80%) and nasal/perinasal skin (3; 60%). Extensive erosions and ulcers were also reported in the axillae (1; 20%), abdomen (1; 20%) and inguinal region (2; 40%). Pruritus was not reported in any of the dogs, and systemic signs such as fever, lethargy and/or anorexia were seen in two dogs (40%) [7].

#### Histopathology

Like in other AISBDs, dermo-epidermal separation leading to vesicle formation was a typical finding in JEBA for the five dogs reported [7]. Formed vesicles were devoid of any inflammation (5/5; 100%) or contained variable numbers of neutrophils (3/5; 60%) and/or eosinophils (2/5; 40%). Superficial dermal, perivascular to interstitial inflammation was mild to moderate and of similar cell type as that seen in vesicles [7]. A positive PAS stain on the dermal side (floor) of the blister could help to differentiate JEBA from EBA (Fig. 4); however, the sensitivity of this test is limited due to the BMZ degradation during the blistering process. Indeed, none of the tested dogs had detectable PAS staining at the BMZ. Similarly, immunohistochemical staining with anti-collagen IV antibody could be used to differentiate JEBA from EBA. In dogs with JEBA, positive collagen IV staining is expected to be at the dermal side (floor) of the blister; however, like the PAS, the sensitivity of this test may be limited. Indeed, positive immunostaining for collagen IV was visible at the dermal side of the blister in three of four tested dogs with JEBA, while no positive staining at the BMZ was visible in the fourth dog [7].

#### Immunopathology

Tissue-bound IgG at the BMZ were detected in two of the four tested dogs (50%) and C3 in one of the three tested dogs (30%) with JEBA. Circulating anti-BMZ IgG and IgA were detected using salt-split canine buccal mucosa tissue in five (100%) and two (40%) tested dogs with JEBA, respectively. These autoantibodies stained the dermal (2/5; 40%) or both dermal and epidermal (3; 60%) side of the salt-split induced blister (Figs. 3 and 4). All five dogs were found to have serum IgG specific for two (alpha-3 and beta-3) or three (with gamma-2) of the three laminin-332 chains [7]; autoantibodies against the classic EBA antigen (NC1 domain of type VII collagen) were not detected.

#### Treatment and outcome

The current information about treatment and outcome of canine JEBA comes from the five described dogs [7]. A spontaneous remission of signs was not reported. Three dogs received glucocorticoid monotherapy, while two received a combination of glucocorticoids and azathioprine. A complete remission of skin lesions was achieved in three of these dogs, two that received glucocorticoid monotherapy and one treated with a combination therapy. Only partial remission was achieved in the two remaining dogs; one dog died from presumed treatment-associated adverse effects and the other was euthanised due to an inability to induce disease remission. In two of the three dogs in which complete remission was achieved, all medications were discontinued without recurrence of the disease.

#### Summary

Canine JEBA is a naturally occurring AISBD with laminin-332 autoantibodies; it affects mucosae, mucocutaneous junctions as well as haired skin. Footpad sloughing is frequent. The acute onset of a severe blistering disease affecting mucosae, mucocutaneous junctions, haired skin and footpads, and a relatively lower age of onset make JEBA nearly identical to classic EBA. Moreover, severe cases of BP may exhibit overlapping clinical features. Therefore, a definitive diagnosis of JEBA cannot be made based on clinical and histological appearance only.

Because advanced immunotesting for anti-laminin-332 autoimmunity is not available in veterinary medicine, additional tests may help to differentiate the diagnoses. PAS staining and/or collagen IV IHC of biopsied blisters, if available, could assist in distinguishing JEBA (dermal staining) from EBA (epidermal staining) but these tests are unable to differentiate JEBA from resembling, collagen XVII-targeting AISBDs. To distinguish JEBA from those diseases, indirect IF using a salt-split buccal mucosa tissue from a healthy dog is required (Fig. 3). In JEBA, anti-BMZ IgG were found to bind to the dermal or both dermal and epidermal aspect of the artificial salt-split blister.

From the handful of available canine JEBA cases, the disease could be successfully controlled in most of them using standard immunosuppressive treatment involving glucocorticoid monotherapy or combination therapy composed of glucocorticoid and a non-steroidal immunosuppressant, azathioprine being the most commonly reported [7]. Once in remission, the dosages of used medications can be markedly reduced or even stopped completely without a subsequent relapse.

### Junctional epidermolysis bullosa acquisita in other animal species

A naturally occurring JEBA has not been reported in any other animal species.

## Mixed autoimmune subepidermal blistering disease (mixed AISBD)

Mixed AISBD is a rare AISBD recognised in dogs in which autoantibodies targeting laminin-332 as well as collagen VII are detected [7]. People with similar clinical, histological and immunological findings have been reported historically [57–59].

### Canine mixed autoimmune subepidermal blistering disease

#### Epidemiology and signalment

Mixed AISBD is a rare autoimmune skin disease in dogs (4% of all AISBDs) and only three cases have been reported to date, each of a different breed (Scottish terrier, Weimaraner, Labrador retriever) [2, 7]. The median age of onset was 3 years (range: 2.5–4 years), and there were two males and one female [7].

#### Clinical signs

Tense vesicles and bullae were localised on haired skin as well as mucosae and/or mucocutaneous junctions. Neck, trunk and/or extremities exhibited lesions in all three dogs. Mucosal and/or mucocutaneous junction involvement was also observed in all three dogs and included oral/labial (3/3; 100%), nasal/perinasal (1; 33%), genital (1; 33%) and periocular and conjunctival (1; 33%) areas [7]. Lethargy was reported in one dog and none of the dogs were pruritic.

#### Histopathology

Like in other AISBDs, dermo-epidermal separation leading to vesicle formation was a typical finding in mixed AISBD in the three dogs reported [7]. Formed vesicles contained variable numbers of neutrophils (3/3; 100%) and eosinophils (3/3; 100%). Similar inflammation of variable intensity could be appreciated in the superficial dermis near the dermo-epidermal separation. None of the two tested dogs had detectable PAS staining at the BMZ and immunohistochemical staining for collagen IV performed in one dog labelled the dermal side (floor) of the blister.

#### Immunopathology

Tissue-bound IgG, IgA and C3 at the BMZ were detected in two (67%), one (33%) and one (33%) of the three tested dogs with mixed AISBD, respectively. Circulating anti-BMZ IgG were detected using salt-split canine buccal mucosa tissue in all three dogs (100%) in which they stained the dermal side (3/3; 100%) of the salt-split induced blister (Figs. 3 and 4). All three dogs were found to have serum IgG that recognised laminin-332 chains (as in JEBA), as well as the NC1 segment of collagen VII (as in EBA) [7].

#### Treatment and outcome

Treatment and outcome information for canine mixed AISBD comes from the three reported dogs [7]. A spontaneous remission was not observed. One dog received glucocorticoid monotherapy, while two received a combination of glucocorticoids and doxycycline (4 mg/kg twice daily) or glucocorticoids and colchicine (0.02 mg/kg/day). A complete remission of erosions and ulcers was achieved in both dogs that received combination therapy. Glucocorticoid monotherapy in one dog was able to control the formation of new lesions, but a complete remission was not achieved. Information on the long-term treatment outcome was not available for any of the dogs, as they were either lost to follow up (2) or euthanatized due to treatment cost (1).

#### Summary

Canine mixed AISBD is a naturally occurring autoimmune disease with laminin-332 and collagen VII autoreactivity and with lesions affecting haired skin as well as mucosae and mucocutaneous junctions. While footpad sloughing has not been described, descriptions are of a limited number of cases and the full spectrum of clinical lesions may not be fully known. Clinical or histological features are not sufficiently unique to allow differentiation of mixed AISBD from other acquired autoimmune blistering diseases. Moreover, neither PAS, anti-collagen IV nor salt-split indirect IF staining is specific enough to confirm the diagnosis. Unfortunately, the advanced immunotesting that is needed to specifically identify the mixed target autoantigens and confirm the diagnosis is not currently available in veterinary dermatology.

Information about the treatment and follow up is limited to only a few cases of mixed AISBD. Two of the three dogs responded to immunosuppressive treatment involving glucocorticoids in combination with doxycycline or colchicine.

### Mixed AISBD in other animal species

A naturally occurring mixed AISBD has not been reported in any other animal species.

## Linear IgA disease (LAD, or linear IgA bullous dermatosis (LABD))

Linear IgA disease is a rare AISBD with autoantibodies (IgA) that target a soluble 120 kDa antigen and/or 97 kDa antigen; both being proteolytic cleavage products of the extracellular domain of BP180 (collagen XVII). In people, the typical clinical presentation is an annular formation of small vesicles (a string of pearls); however, diverse clinical features overlapping with other AISBDs have been reported [60]. Therefore, immunopathological features have been proposed as a diagnostic criterion in people. Unfortunately, diagnostic criteria of LAD remain confusing, and it remains unclear whether the diagnosis of LAD should be made if exclusively IgA deposits are detected along the BMZ, or if they are detected together with other immunoglobulins (IgG, IgM, IgE, etc.) [61]. Indeed, people with linear deposition of both IgA and IgG autoantibodies at the BMZ have been recently reported under the diagnosis of linear IgA/IgG bullous disease (LAGBD) [62].

### Canine linear IgA disease

#### Epidemiology and signalment

Linear IgA disease is a rare AISBD described in only two adult dogs (3% of all AISBDs); one cross-bred Labrador retriever (3-year-old, female spayed) and one briard (4-year-old, male neutered) [2, 5]. Interestingly, human LAD exhibits a biphasic age distribution affecting both young children (age 6 months to 6 years) and older adults (> 60 years) [60, 63].

#### Clinical signs

One of the two reported dogs exhibited erosive and ulcerative lesions that resembled a canine EBA (oral cavity, pinnae, friction areas of axillae and abdomen and footpads), while in the other dog only nasal planum, oral cavity and footpads were affected [5].

#### Histopathology

Like in other AISBDs, dermo-epidermal separation leading to vesicle formation was found in 2 dogs with LAD [5]. Vesicles were devoid of inflammatory cells or contained a few neutrophils. There were no intact or degranulated eosinophils in the vesicles or in the superficial dermis of the two dogs. Immunostaining of skin biopsy sections from both dogs for collagen IV showed positive staining at the dermal side (floor) of the blisters, thus supporting the separation through the lamina lucida of the BMZ.

#### Immunopathology

Tissue-bound IgA and IgG along the BMZ were detected in one (50%) and two (100%) dogs with LAD, respectively. Circulating anti-BMZ IgA and IgG that bound to the epidermal side (roof) of the artificial cleft on a salt-split mucosal substrate were visualised in both dogs.

Immunoblotting studies established that these IgA and IgG recognised the cleaved, soluble 120 kDa antigen (LAD-1 antigen) present in a keratinocyte culture-conditioned medium, but not in cell membrane-rich fractions (i.e., it represents the processed extracellular domain of collagen XVII) [5].

#### Treatment and outcome

Treatment and outcome information are not available for the two reported dogs. In people, treatment approaches depend on the disease aetiology, severity and response to treatment. In drug-induced LAD, vancomycin being the most common trigger, discontinuation of the drug with or without concurrent medical management is recommended. The drug of choice for spontaneous LAD in people are sulfones, dapsone being the most common one, or colchicine [60]. In cases developing dapsone-associated side effects or lacking a sufficient response, a combination of glucocorticoids and non-steroidal immunosuppressant such as azathioprine, mycophenolate or cyclosporine may be necessary. Similar treatment approaches could be considered in dogs with LAD.

#### Summary

Canine LAD is a naturally occurring AISBD with IgA (+/− IgG) reactivity against soluble antigens of collagen XVII. Its clinical and histological features are overlapping with other AISBDs and, therefore, the diagnosis confirmation is dependent upon the demonstration of a linear and homogeneous deposit of IgA along BMZ within the skin biopsy section (direct IF) or by confirmation of circulating anti-BMZ IgA antibodies binding to the epidermal side (roof) of the salt-split blister (indirect IF).

Because of the lack of information about treatment and outcome in canine LAD, dogs with confirmed or suspected LAD should be treated in a similar manner as dogs with other AISBDs.

### Linear IgA disease in other animal species

A naturally occurring LAD has not been reported in any other animal species.

## Pemphigoid gestationis (PG)

Pemphigoid gestationis is an AISBD affecting women, usually during the 2nd or 3rd trimester of their pregnancy. Additionally, PG has been reported in women with trophoblastic tumour or choriocarcinoma [64]. In people, PG is clinically, histologically, and immunologically similar to BP, and resolves spontaneously 4–14 weeks post-partum [64].

### Canine pemphigoid gestationis

#### Epidemiology and signalment

The information about PG in veterinary dermatology is based on a single unpublished case of a 4-year-old pregnant female Tibetan spaniel, which was submitted to the authors’ immunodermatology laboratory for further testing.

#### Clinical signs

**Fig. 14:**
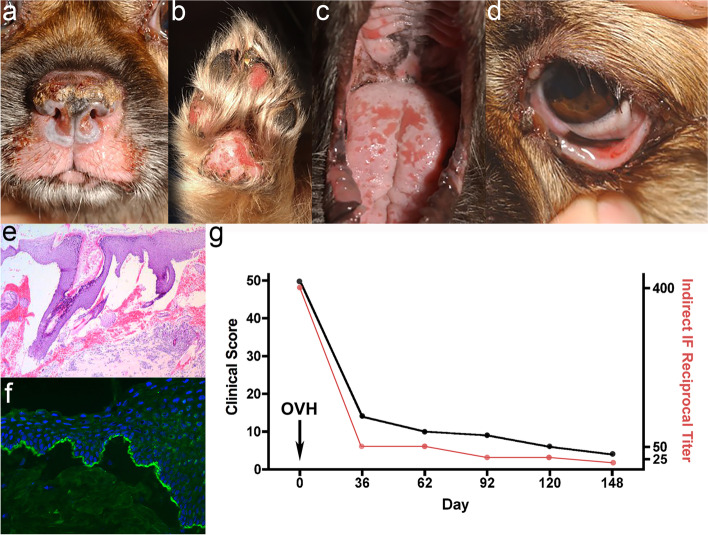
Pemphigoid gestationis in a dog: Deep erosions affecting the nasal planum and muzzle, footpads, eyelids and oral cavity (**a-d**), **e** histopathology of canine PG depicting dermo-epidermal separation, **f** indirect immunofluorescence on salt-split buccal mucosa demonstrated IgG along the roof of the blister, **g** graphic correlation of clinical signs severity and circulating anti-basement membrane zone IgG titres (courtesy of Dr. Thierry Olivry). Clinical and histological photos courtesy of Dr. Jan Rybnicek

Four and half weeks into the pregnancy, the dog presented with erosions and ulcers on mucosae/mucocutaneous junctions (conjunctiva, gingival mucosa, tongue, hard palate, vulva, and nasal planum) as well as haired skin (perinasal, periocular and lip areas, concave pinnae, nipples, and footpads) (Fig. 14a-d). Intact vesicles were observed on the lips and buccal mucosa. Lethargy and reduced appetite were reported.

#### Histopathology

Dermo-epidermal separation leading to vesicle formation was present in all biopsy samples (Fig. 14e) from one dog (unpublished case; JR and TO). Large subepidermal clefts commonly extended to involve hair follicle infundibula and even sebaceous gland ducts and lobules. Vesicles were devoid of inflammatory cells or contained neutrophils, but vesicle rupture limited assessment of vesicle contents. Mixed, neutrophilic, dermal inflammation was mild, superficial, and perivascular. Several biopsies contained moderate epidermal hyperplasia. Hypergranulosis, possible granular layer separation, and occasional shallow erosions with a few neutrophils were present. Because of its rarity, the variation of histopathology changes is not well known.

#### Immunopathology

Tissue-bound IgG, IgM and C3 along the BMZ were detected in submitted skin biopsy samples. In areas with a dermo-epidermal separation, the deposit was on the epidermal side (roof) of the formed blister. Circulating anti-BMZ IgG bound to the epidermal side of the artificial cleft on a salt-split mucosal substrate (Fig. 14f). These findings suggested BP180 (collagen XVII) to be the most likely target antigen. Further confirmation of the target antigen was not performed.

#### Treatment and outcome

All skin lesions started to heal shortly after an ovariohysterectomy was performed, and complete remission was achieved within several months. This correlated with a gradual reduction and eventual disappearance of the anti-BMZ IgG (Fig. 14g). No immunosuppressive therapy was necessary.

#### Summary

Canine PG is a very rare AISBD with current information limited to a single unpublished case. Therefore, the full spectrum of clinical lesions may not be fully known. The diagnosis of PG should be based on confirmation of pregnancy in a dog with skin lesions clinically and histologically compatible with AISBD.

Considering the single known case and the major target antigen in people (BP180; collagen XVII), skin biopsies stained with PAS or anti-collagen IV antibody should demonstrate positive staining on the dermal side (floor) of the blister. Sensitivity of these tests in canine PG is unknown. If available, indirect immunofluorescence using salt-split buccal mucosa tissue can be used to demonstrate anti-BMZ IgG (+/− other classes) along the epidermal side (roof) of the artificial salt-split blister. Available information about treatment of canine PG is also very limited. In this single case, no immunosuppressive treatment was required, and spontaneous resolution of the disease was observed after an ovariohysterectomy. In people, treatment depends on the disease severity and involves bathing and topical emollients, topical glucocorticoids or, in severe cases, low dose of systemic glucocorticoids (0.3–0.5 mg/kg/day). A spontaneous remission of the disease is observed within weeks to a few months postpartum [64].

### Pemphigoid gestationis in other animal species

A naturally occurring PG has not been reported in any other animal species.

## Type-1 bullous systemic lupus erythematosus (BSLE-I)

Type-1 bullous SLE is a disease in which a patient suffering with SLE develops signs compatible with AISBD and autoantibodies targeting BMZ proteins. The most common mechanism proposed in BSLE-I is intermolecular epitope spreading with the subsequent development of anti-collagen VII autoantibodies, which have been demonstrated in people as well as dogs. In addition to the anti-collagen VII autoreactivity, multiple other target antigens have been uncovered in people (laminin-332, laminin-311, BP230, etc.) [65].

### Canine type-1 bullous systemic lupus erythematosus

#### Epidemiology and signalment

Bullous SLE-I is an autoimmune skin disease with only one case, a 4-year-old male bichon frise, described in the literature [66]. As such, it is very rare AISBD in this species (1% of all AISBDs) [2].

#### Clinical signs

The dog suffered concurrently with SLE and AISBD characterised by erosions, ulcers and crusts on the haired skin (elbow, axillae, lateral thorax, concave pinnae), mucosae and mucocutaneous junctions (oral cavity, labial commissures) as well as footpads [66].

#### Histopathology

Skin biopsy samples from the single BSLE-I affected dog revealed dermo-epidermal separation leading to vesicle formation, which extended to hair follicles in some locations. Vesicles were devoid of inflammatory cells or contained neutrophils and histiocytic cells. Interface dermatitis and basal cell apoptosis were not observed. Superficial, dermal perivascular to interstitial mixed inflammation was present in some areas of blister formation but was absent in others [66].

#### Immunopathology

Tissue-bound IgG and complement (C3) were detected along the BMZ. In areas with blister formation, these deposits were on the dermal side (floor) of the dermo-epidermal separation. Circulating anti-BMZ IgG that bound to the dermal side of the artificial cleft on a salt-split mucosal substrate were detected by indirect IF. These circulating IgG were demonstrated to bind the NC1 domain of collagen VII [66].

#### Treatment and outcome

Treatment with 4 mg/kg/day of prednisone for 3 weeks failed to induce lesion remission. Addition of dapsone (1 mg/kg three times daily) resulted in improvement of skin lesions and clinical laboratory abnormalities associated with the SLE; however, due to a treatment-refractory recurrence of the skin lesions 5 months later, euthanasia was elected [66].

#### Summary

Canine BSLE-I is a very rare AISBD, for which the diagnosis is based on confirmation of SLE and the concurrent presence of skin lesions that are clinically and histologically compatible with AISBD. Skin biopsy sections stained with PAS or anti-collagen IV antibody should demonstrate positive staining on the epidermal side (roof) of the blister, supporting the separation below lamina densa in the BMZ in case of anti-collagen VII autoimmunity; however, as other BMZ autoantigens have been confirmed in people, other staining patterns may be observed [65].

### Type-1 bullous SLE in other animal species

A naturally occurring bullous SLE has not been reported in any other animal species.

## Summary

Autoimmune subepidermal blistering diseases are rare diseases recognised in animals. Reports of dogs with AISBDs are more frequent than those of other species, and, therefore, most of our knowledge about these animal diseases is extrapolated from this species. The clinical presentation and histological confirmation of subepidermal clefting are required to make a diagnosis of AISBD, but our current ability to differentiate individual AISBDs is limited.

The typical clinical presentations include tense vesicles and/or bullae, which rapidly progress into deep erosions and ulcers. In most AISBDs, lesions can be found on haired skin, as well as mucosae and mucocutaneous junctions and the clinical phenotype can overlap between many of these AISBDs. An exception is MMP in which lesions are almost exclusively found on mucosae and/or mucocutaneous junctions. This allows clinicians to diagnose MMP solely based on clinical (acquired, blistering disease affecting mucosae and/or mucocutaneous junctions) and histological features.

In people, BP often presents without mucosal lesions, which is used as an important diagnostic feature for BP. The sensitivity and, more importantly, the specificity of this observation in animals remain unknown. Indeed, while a marked haired skin involvement was seen in animals with BP, one must bear in mind that many dogs (67%), cats (50%) and all horses (100%) with BP exhibited mucosal and mucocutaneous lesions. Therefore, BP in these species can clinically and histologically overlap with other AISBDs. A relevant clinical information such as concurrent SLE in BSLE-1 or pregnancy in PG, can allow for a specific diagnosis to be achieved; these conditions are very rare, however.

Histological features that differentiate individual AISBDs have not been identified. Some features, such as the presence of eosinophils in lesions, which were proposed to be specific for BP, have been identified in other AISBD subtypes and, therefore, cannot be used reliably to differentiate between the individual AISBDs. Others, such as a rowing of neutrophils along the BMZ and an absence of inflammation, may appear to be more specific (e.g., EBA and MMP, respectively), but because of the limited number of available biopsies from other AISBDs, the specificity of these features remain unknown.

The next helpful diagnostic step to further distinguish individual AISBDs is to demonstrate the depth of the dermo-epidermal separation. Specific tests include PAS stain, anti-collagen IV IHC or IF, and indirect immunofluorescence using patient’s serum and salt-split tissue for testing (Figs. 3 and 4). Although these tests are not difficult to perform, their sensitivity can be reduced due to the BMZ proteolytic degradation following the granulocyte-rich inflammation and enzymatic cascade involved in the blistering process. Additionally, while most cases may exhibit a staining pattern matching the expected immunophenotype, differences in staining patterns have been reported in the literature, thus reducing the specificity of these tests. Most of these tests are currently not offered by commercial laboratories. Therefore, the diagnostic ability of veterinarians to properly diagnose individual AISBDs, except MMP, BSLE-I and PG, is limited. This inability limits our progress in gathering more information about these rare skin diseases, such as unique clinical and histological features, treatment response to a specific drug or drug combinations, existing co-morbidities, tendency to undergo long-term remission off drugs, etc. Therefore, presently, treatment recommendations for animals affected with different AISBDs are limited to the following general immunosuppression principles: i) the achievement of a rapid disease remission using fast-acting drugs such as immunosuppressive dose of oral glucocorticoids (with or without non-steroidal immunosuppressive drugs), and ii) a safe maintenance of disease remission using, usually, non-steroidal immunosuppressive drugs. The latter include tetracycline antibiotics and niacinamide, especially in MMP, colchicine in EBA or, in poorly responsive or severe cases, azathioprine, ciclosporin, mycophenolate, oclacitinib, leflunomide or other.

## Supplementary Information

**Additional file 1.**

## Data Availability

This article being a review of published information, data sharing is not applicable, as no datasets were generated or analysed.

## Abbreviations

AISBDs: autoimmune subepidermal blistering diseases
BMZ: basement membrane zone
BP: bullous pemphigoid
BSLE-1: bullous systemic lupus erythematosus type 1
EBA: epidermolysis bullosa acquisita
IHC: immunohistochemistry
IF: immunofluorescence
JEBA: junctional epidermolysis bullosa acquisita
LAD: linear IgA dermatosis
MMP: mucous membrane pemphigoid
PAS: periodic acid-Schiff
PG: pemphigoid gestationis
